# Nanovehicles as a novel target strategy for hyperthermic intraperitoneal chemotherapy: a multidisciplinary study of peritoneal carcinomatosis

**DOI:** 10.18632/oncotarget.4309

**Published:** 2015-05-27

**Authors:** Maciej Nowacki, Marek Wisniewski, Karolina Werengowska-Ciecwierz, Katarzyna Roszek, Joanna Czarnecka, I. Łakomska, Tomasz Kloskowski, Dominik Tyloch, Robert Debski, Katarzyna Pietkun, Marta Pokrywczynska, Dariusz Grzanka, Rafał Czajkowski, Gerard Drewa, A. Jundziłł, Joseph K. Agyin, Samy L. Habib, Artur P. Terzyk, Tomasz Drewa

**Affiliations:** ^1^ Chair of Regenerative Medicine, Tissue Engineering Department, Ludwik Rydygier's Collegium Medicum in Bydgoszcz Nicolaus Copernicus University, Torun, Poland; ^2^ Physicochemistry of Carbon Materials Research Group, Faculty of Chemistry, Nicolaus Copernicus University in Torun, Poland; ^3^ Invest-Tech, Research and Development Center, Torun, Poland; ^4^ Department of Biochemistry, Faculty of Biology and Environment Protection, Nicolaus Copernicus University in Torun, Poland; ^5^ Faculty of Chemistry, Nicolaus Copernicus University in Torun, Poland; ^6^ Department of Pediatric Hematology and Oncology, Ludwik Rydygier's Collegium Medicum in Bydgoszcz Nicolaus Copernicus University in Torun, Poland; ^7^ Chair of Dermatology Department, Faculty of Medicine, Nicolaus Copernicus University, Toruń, Sexually Transmitted Diseases and Immunodermatology, Bydgoszcz, Poland; ^8^ Department of Medical Biology, University of Bydgoszcz, Poland; ^9^ Department of Biochemistry, The University of Texas Health Science Center, San Antonio, TX, USA; ^10^ Department of Cellular and Structural Biology, The University of Texas Health Science Center, San Antonio, TX, USA; ^11^ Department of Geriatric, South Texas Veterans Health System, The University of Texas Health Science Center, San Antonio, TX, USA; ^12^ Urology Department, Nicolaus Copernicus Hospital in Torun, Torun, Poland

**Keywords:** carcinomatosis, palliative, hyperthermic intraperitoneal chemotherapy, intraperitoneal perfusion and nanovehicles

## Abstract

In general, detection of peritoneal carcinomatosis (PC) occurs at the late stage when there is no treatment option. In the present study, we designed novel drug delivery systems that are functionalized with anti-CD133 antibodies. The C1, C2 and C3 complexes with cisplatin were introduced into nanotubes, either physically or chemically. The complexes were reacted with anti-CD133 antibody to form the labeled product of A0-o-CX-chem-CD133. Cytotoxicity screening of all the complexes was performed on CHO cells. Data showed that both C2 and C3 Pt-complexes are more cytotoxic than C1. Flow-cytometry analysis showed that nanotubes conjugated to CD133 antibody have the ability to target cells expressing the CD133 antigen which is responsible for the emergence of resistance to chemotherapy and disease recurrence. The shortest survival rate was observed in the control mice group (K3) where no hyperthermic intraperitoneal chemotherapy procedures were used. On the other hand, the longest median survival rate was observed in the group treated with A0-o-C1-chem-CD133. In summary, we designed a novel drug delivery system based on carbon nanotubes loaded with Pt-prodrugs and functionalized with anti-CD133 antibodies. Our data demonstrates the effectiveness of the new drug delivery system and provides a novel therapeutic modality in the treatment of melanoma.

## INTRODUCTION

Peritoneal carcinomatosis (PC) presents a significant challenge within current surgical oncology. PC refers to a variety of organ-based malignancies in the peritoneal cavity resulting from uncontrolled and rapidly progressing metastatic processes [1-3]. PC has a very poor prognosis and it is invariably terminal [4-7]. Indeed, there is a serious lack of treatment options for patients suffering from advanced-stage PC. The combination of palliative cytoreductive surgery (CRS) and hyperthermic intraperitoneal chemotherapy (HIPEC) are promising and significantly improve the patient's quality of life. However, these combinations are characterized by a high in-hospital mortality rate and short periods of survival [8-9].

Nanotechnology has had a significant impact on medicine [10-12]. The application of nanotechnology in melanoma treatment highlights several nanoparticle-delivered drugs approved by the US Food and Drug Administration, which are currently in clinical trials [13]. Low molecular weight drugs such as cisplatin or *cis*-diamminedichloroplatinum (CDDP) have been shown to accumulate in cancerous tissue whilst quickly penetrating the circulatory system. Such drug delivery systems subsequently increase the therapeutic effect of platinum complexes by enhancing cytotoxicity within the tumor due to their slow release. Novel drug delivery-systems (DDS) that are based on different types of nanoparticles can potentially act as nano-containers for targeted anti-cancer treatment. Carbon nanotubes (CNTs) are among the most frequently used DDS [14-16].

Direct targeting of cancerous tissues with anti-cancer drugs can be achieved via several strategies. Typically, antibodies are directed towards specific surface antigens or receptor proteins within tumor cells enabling drugs or drug carriers to be delivered to the target [17]. The nano-material is functionalized with a specific recognition mechanism such as those decorated with antibodies, giving rise to new and efficient delivery systems for both locally or systemically administered drugs [18]. Indeed, the development of functionalized nano-materials is one of the driving forces in the recent development of new materials classes for applications in biology and medicine. In particular, CNTs, with their unique physical and chemical properties hold great promise for drug delivery and have been used to directly target tumors in the last few years [19-23]. CNTs have water solubility and biocompatibility properties and are able to cross cell membranes, shuttling a wide range of biologically active molecules into cells [24-28]. CNTs have excellent stability in the aqueous phase and can be bioconjugated to targeting ligands such as antibodies and peptides. These novel DDS are able to recognize specific cell receptors and provide targeted CNT bioconjugates, which are useful for biological sensing as well as imaging [29-34].

In the present study, we determined whether selected nanovehicles based on anti-CD133 antibodies bioconiugated to carbon nanotubes loaded with platinum (Pt) -prodrugs could be successfully used as targeted drug delivery systems in modified hyperthermic intraperitoneal chemotherapy without perfusion. New anti-cancer complexes, namely cis-[PtCl_2_(dbtp)_2_] (labeled C2), where dbtp is 5,7-ditertbutyl-1,2,4-triazolo[1,5-a]pyrimidine, and [Pt(C_4_H_4_O_5_)(dbtp)_2_] (labeled C3), have been synthesized and their efficacy compared to unmodified CDDP (C1). In addition, we tested whether nanovehicles can prolong survival via induced peritoneal carcinomatosis (PC) in mice. We proposed to establish drug delivery system that is more efficient and present the most significant inhibiting effect on melanoma cell growth induced by PC in a mouse model.

## RESULTS

### Thermal analysis of prodrugs and drug delivery systems

Thermal analysis results for pure C2 (/C3) and for A0-o, A0-o-C2 (/C3)-phys, A0-o-C2 (/C3)-chem-n, A0-o-C2 (/C3)-chem are displayed in Figure 2. For the both complexes after drug deposition, we observed higher thermal stability (comparing to the drug alone) of C2 (/C3) on nanotubes. The comparison of thermogravimetric curves determined for pure C2 and C3 drugs shows lower thermal stability of the latter. This can be caused by the different nature of the Pt (II) - ligand bonds. Since both prodrugs and dbtp are common ligands, one can conclude that the differences in drugs thermal stability are caused by the nature of the bonds between the central ion and two other ligands. In the case of the C2 complexes, the ligand is bonded to the central Pt(II) ion by Cl(I) while in C3 the O(II)-Pt(II) bonds occur. Following the HSAB theory since O(II) is a hard base, and Pt(II) is a soft acid one can expect weaker bond than for Pt(II) - Cl(I).

In general, lower thermal stability is observed for nanotubes after drug deposition. This is probably due to catalytic activity of Pt in nanotubes decomposition. More importantly, the mass loss of the A0-o-C2-phys and A0-o-C3-phys samples corresponded to the 2:1 C2 (/C3): A0-o mass ratio after burning. However, additional purification in DMF (or acetone) was necessary to remove physically adsorbed drugs from chemical bonded complexes (A0-o-C2(/C3)-chem-n and A0-o-C2 (/C3)-chem).

**Figure 1:**
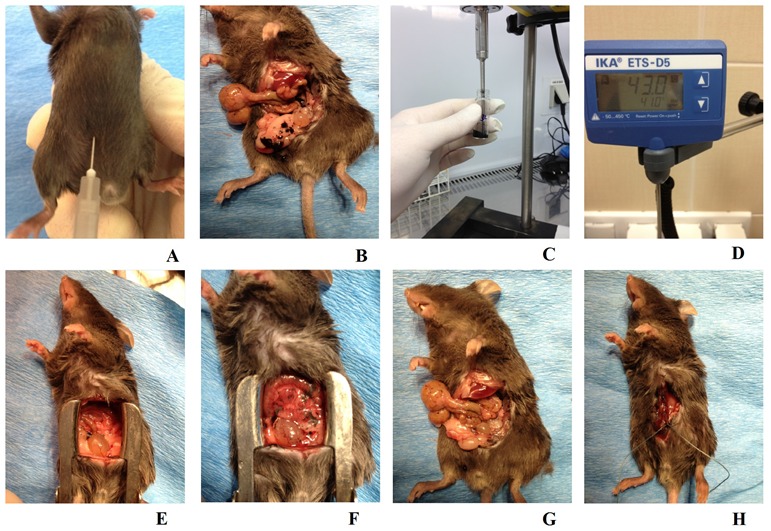
Animal model of peritoneal carcinomatosis and chemotherapy procedures **A.** To induce peritoneal carcinomatosis, the 1 × 10^6^ B16 cells were injected into the right lower abdomen quadrant of all animals from each group. **B.** Intraoperative picture of a mouse from the K3 group, one week after the B16 cells had been injected, the PC process at an advanced stage and exposed following preparation of the peritoneal and visceral tissue. **C.** Nanovehicles (DDS) were sonicated in 0.7 ml of sterile PBS for three minutes to obtain a homogenous solution. **D.** Continuous checking ensured that a consistent temperature was maintained across all study groups. **E.** A mouse from the A0-o-C1-chem-CD133 group just before the modified HIPEC procedure was performed. **F.** The same mouse as in E immediately after the application of liquid chemotherapy. **G.** The same mouse as in B just after the surgical cytoreduction of tumors. **H.** The same mouse as in E/F having sutures applied immediately after the experimental HIPEC procedure, a process consistently applied across all study and control groups.

**Figure 2:**
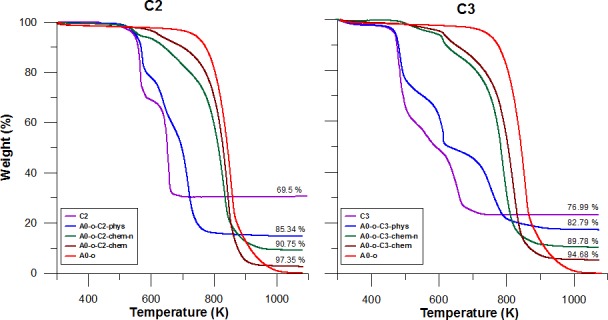
Thermal analysis results for pure C2 (/C3), A0-o, A0-o-C2 (/C3)-phys, A0-o-C2 (/C3)-chem-n, and A0-o-C2 (/C3)-chem

### STEM analysis

To obtain evidence of prodrug activity on the surface of the carbon nanotubes, Z-contrast STEM images were taken showing clusters of CDDP molecules as previously described [35]. Major differences were observed in the drug distribution within the carbon nanotubes attributable to the varying deposition methods used. High-resolution transmission electron microscopy and z-TEM images of the A0-o-C2-phys sample are displayed in Figure 3A and the A0-o-C3-phys sample in Figure 3B. On the one hand, large clusters of C2/C3 on the CNT surfaces could be observed as large light dots, caused by the physical adsorption process. High-resolution transmission electron microscopy and z-TEM images of the A0-o-C2-chem sample are displayed in Figure 4A and the A0-o-C3-chem sample in Figure 4B. On the other hand, clusters of the complexes could not be detected after chemical deposition and the surfaces of CNT appeared foggy with no clear dots visible. Minuscule nanoclusters of C2(/C3) complexes were observed on the tube surfaces and results from TG-analysis showed that the total amount of drugs remained close to 10 wt %. The appearance of only few dots demonstrated that Pt-complexes were more dispersed after chemical deposition and were undetectable.

**Figure 3A:**
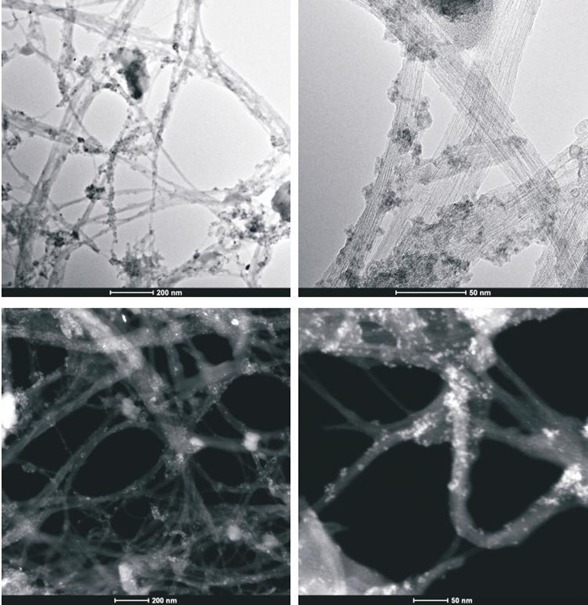
High-resolution transmission electron microscopy and z-TEM images of the A0-o-C2-phys sample

**Figure 3B:**
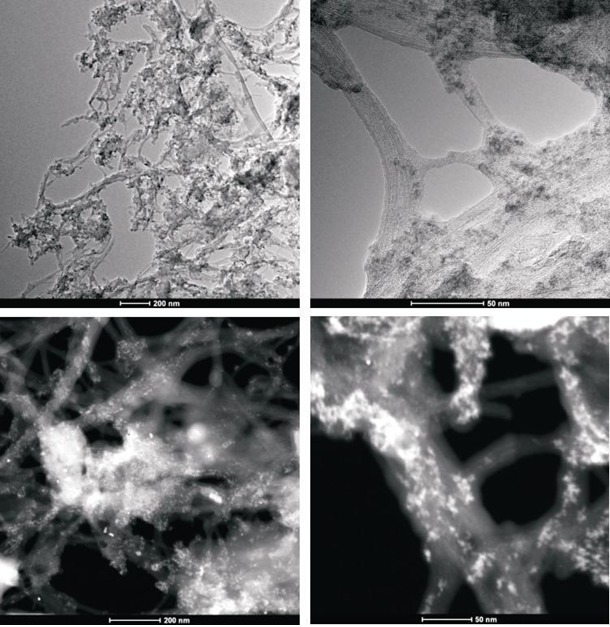
High-resolution transmission electron microscopy and z-TEM images of the A0-o-C3-phys sample

**Figure 4A:**
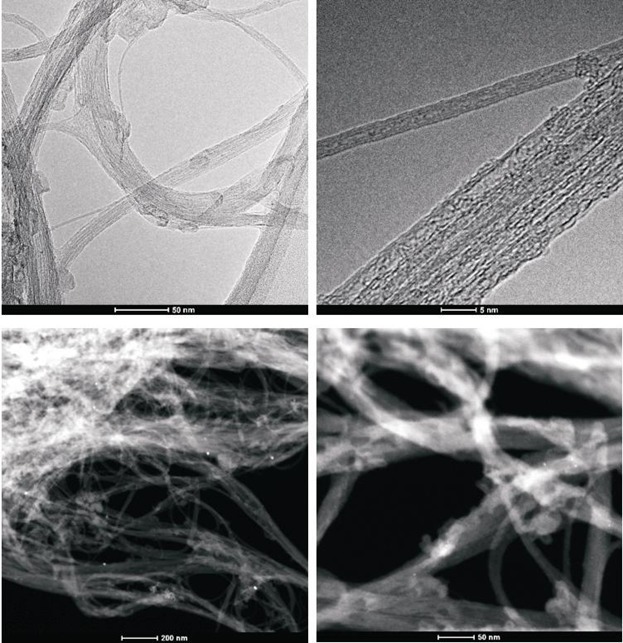
High-resolution transmission electron microscopy and z-TEM images of the A0-o-C2-chem sample

**Figure 4B:**
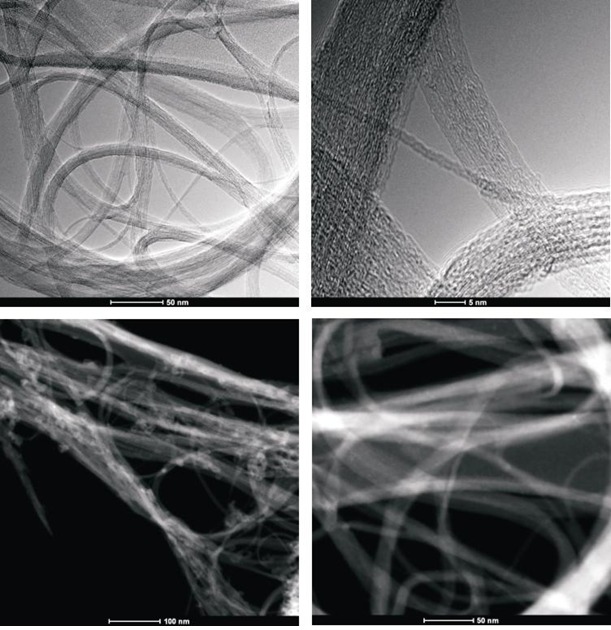
High-resolution transmission electron microscopy and z-TEM images of the A0-o-C3-chem sample

### Nitrogen adsorption - desorption results

**Figure 5:**
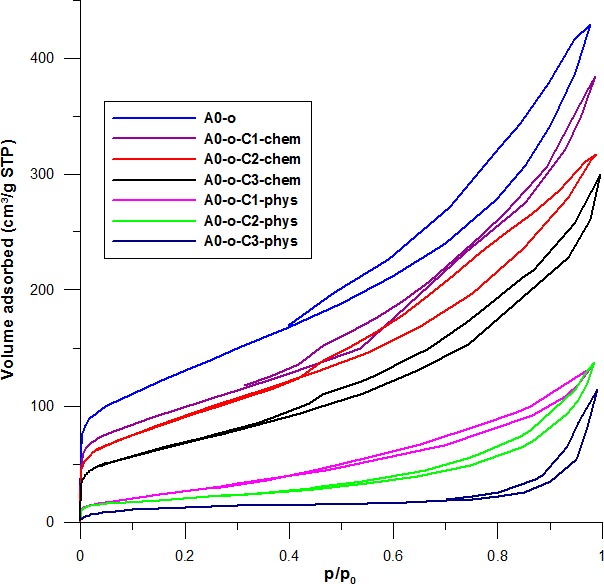
The influence of C2 deposition on nitrogen adsorption-desorption isotherms recorded at a temperature of 77 K for the A0-o, A0-o-C2-phys, and A0-o-C2-chem samples

Low temperature of N_2_ adsorption-desorption isotherms were measured to determine whether there is physical adsorption or chemical bonding in the samples of Pt-complexes. The lower temperature of N_2_ adsorption-desorption isotherms blocks the pores to a higher extent. Data in Figure 5 shows that initially, the nanotubes display high adsorption followed by a decrease after the deposition of drugs permeated the pores. In the case where drugs were physically adsorbed, there was a near disappearance of hysteresis in the isotherm and the adsorption rate decreased from C1 to C2 to C3. This decrease was inversely proportional to the volumes of the complexes (C3 having the largest volume and C1 the smallest). The drastic decrease in the volume of mesopores results from the conductive effect of large amounts of drug clusters located between the nanotubes that block the internal channels.

### Cytotoxicity analyses

**Figure 6:**
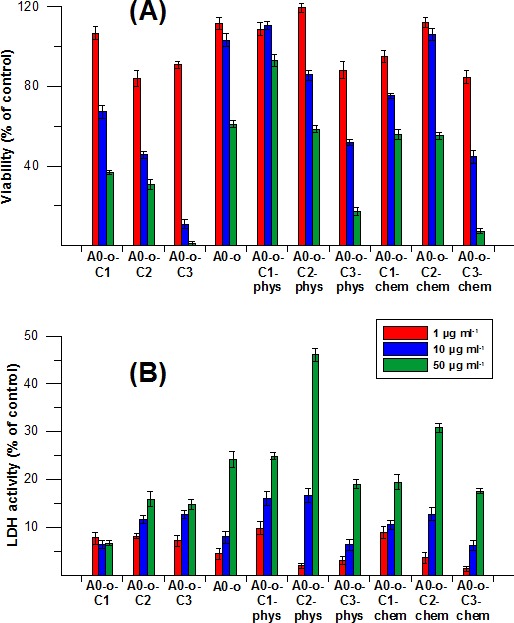
Cytotoxicity of drug delivery systems measured by MTT (A) and LDH (B) assays in CHO cells

Cytotoxicity analyses were performed to evaluate the potential therapeutic application of the drug delivery systems. All prodrug delivery systems (Pt-complexes, nanotubes) were assessed for their cytotoxicity in CHO cells by MTT assay and LDH activity. Data in Figure 6A showed that both the C2 and C3 Pt-complexes are more toxic than that of the C1. The viability of CHO cells treated with different concentrations of C2 and C3 Pt-complexes ranging from 1 μg ml^−1^ and 50μg ml^−1^ was expressed as a percentage of control and decreased from 84% to 31 % and from 91% to 1%, respectively.

In contrast, A0-o appeared to be nontoxic at the highest concentration, whilst the decrease in cell viability was 60% at the lowest concentration due to its cytostatic properties. Meanwhile, A0-o-C1-phys remained non-toxic (with over 90% viability at a concentration of 50 μg ml^−1^), while the C2 and C3 Pt-complexes showed toxicity is concentration-dependent. On the other hand, the highest concentration of A0-o-C3-phys decreased cell viability to below 20%. Interestingly, the chemical bonding of Pt-complexes to the surface of the nanotubes did not cause any significant alterations in their levels of toxicity.

The LDH activity data in Figure 6B showed similar cytotoxic properties and concentration-dependence across all Pt-complexes. Cells treated with Pt-complexes did not reveal any dramatic differences between materials prepared through physical adsorption or chemical bonding. Interestingly, only treatment with the C2 complexes caused excessive increase in LDH activity as a marker of cell membrane damage (Figure 6B).

### Anti-cancer properties in melanoma cells

**Figure 7:**
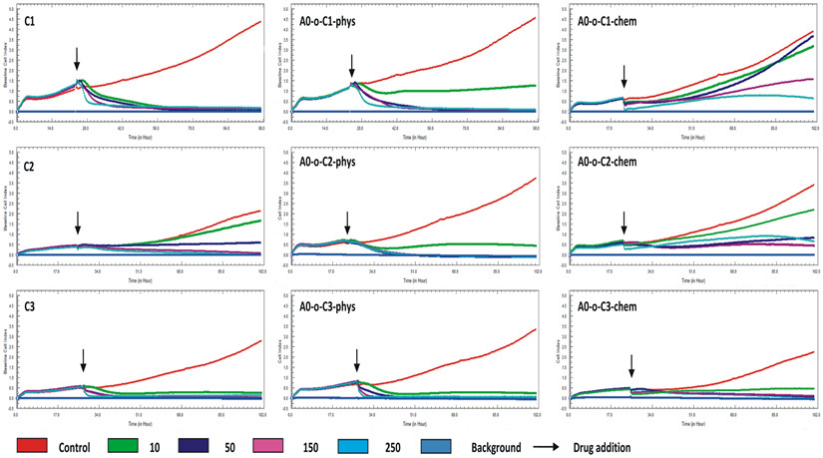
Real time cell analysis of B16-F10 after treatment with drugs administered alone (C1, C2 and C3) and with drugs delivered chemically or physically via nanotubes (A0-o-C1, A0-o-C2 and A0-o-C3)

The anti-cancer properties were tested in B16 melanoma cells. Cell proliferation data for the free drugs and those delivered via nanotubes are shown in Figure 7. Cells treated with C1 and C3 compounds did not show any changes in cell proliferation at low and high concentrations while low concentrations of the C2 complex (10 and 50 μg ml^−1^) showed a decrease in cell proliferation compared to control (Figure 7). In all treatments, the cell index (CI) values remained above the baseline when the lowest (10 μg ml^−1^) concentration was applied with no observable differences at higher concentrations (50 – 250 μg ml^−1^). One the other hand, the drugs that were chemically bonded to nanotubes appeared to have weaker effect on cell proliferation compared to those that were adsorbed physically. One exception was the A0-o-C3-chem complex where a decrease in the CI value compared to the baseline level was observed at longest exposure. The remaining two complexes in this group did not cause a decrease the CI even at the highest concentration (250 μg ml^−1^). The weakest anti-cancer activity was observed in the A0-o-C1-chem complex where 10 and 50 μg ml^−1^ concentrations resulted in the same CI value as the control (Figure 7). Moreover, the increase in DDS concentration led to a decrease in cell viability and possibly increased the rate of cell death. Taken together, the platinum drugs adsorbed physically on nanotubes were found to be the most effective tools against B16 melanoma cells.

**Figure 8:**
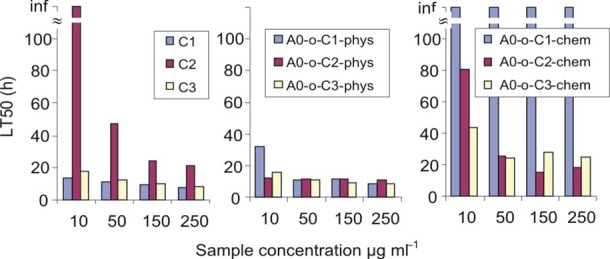
LT50 of tested compounds calculated on the basis of real time cell analysis

The LT50 was calculated on the basis of results obtained from real-time cell analysis (Figure 8). LT50 is defined as the time needed to decrease the number of cells by 50% in comparison to the number of cells present initially (arrows on Figure 7). For the C2 compound, a decrease in the LT50 in the presence of DDS was observed in both chemically and physically modified systems indicating that B16 cells are most sensitive to the C2 compounds. In all cases, a decrease in number of B16 cells was observed in a dose-dependent manner, but A0-o-C1-chem appeared to be completely non-toxic.

### Flow cytometry analysis

**Figure 9:**
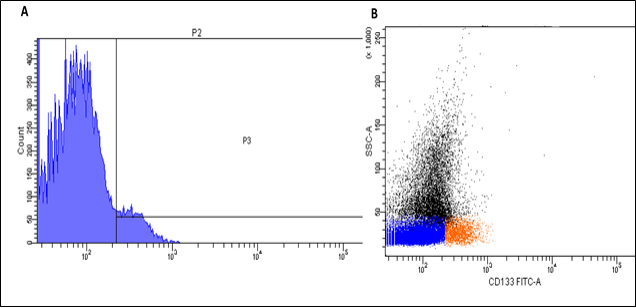
Flow cytometry analysis of the anti-CD133 antibody delivered with nanotubes **A.**– 5.4% of positively labeled cells (gate P3). **B.** – Positively labeled cells (red) are small in size and have low granularity.

We showed that nanotubes attached to the anti-CD133 antibody successfully bound to cells expressing the CD133-antigen using flow cytometry analysis. Around 5.4% of cells (gate P3) showing small-sized cells with low granularity were positively (red) labeled (Figure 9A & 9B). Small positively labeled cells with low granularity were observed indicating that the nanotube-bound anti-CD133 antibody has high affinity for CD133 antigen and binds to stem cells at an early stage of development.

### Survival rate and statistical analysis

**Figure 10:**
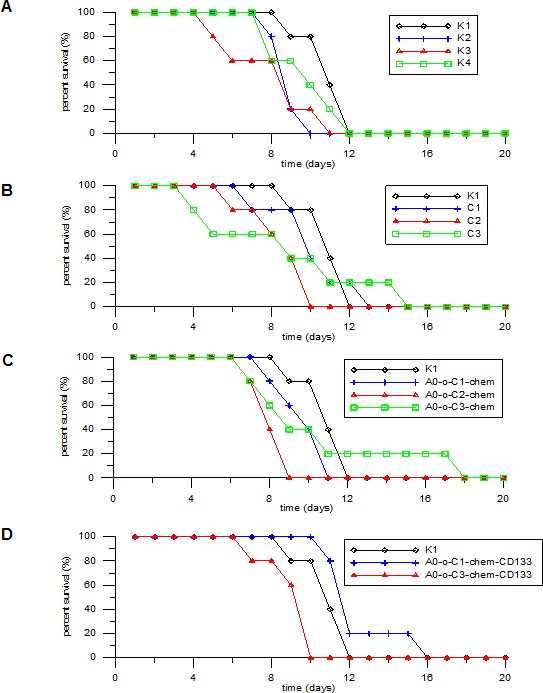
Survival rate analysis **A.** K2;K3;K4 group vs. K1 group. **B.** C1;C2;C3 group vs. K1. **C.** AO-o-C1-chem; AO-o-C2-chem; AO-o-C3-chem group *vs*. K1 group.**D.** AO-o-C1-chem-CD133 and AO-o-C3-chem-CD133 group *vs*. K1 group.

The survival rates at different levels of mortality were evaluated in all eight treated (labeled as: C1, C2, C3, A0-o-C1-chem, A0-o-C2-chem, A0-o-C3-chem, A0-o-C1-chem-CD133, A0-o-C3-chem-CD133) and control groups of mice. The first animal death was noted on the fourth day of the experiment in group C1, whilst the longest individual survival rate was sixteen days-in group A0-o-C1-chem-CD133 (Figure 10). Statistical evaluation and survival median were also calculated. The shortest general survival (8 days) was observed in the control group K3 whereas the longest survival (12.6 days) was observed in group A0-o-C1-chem-CD133. The survival rates for the other control groups were as follows: K1 - 11.0 days; K2 - 9.0 days and K4 - 8.4 days. Statistical analysis using the Kruskal Wallis test revealed significant differences in animal survival rates between the groups (*p* < 0.05). However, Bonferroni corrected pairwise test showed that the only significant difference in animal survival was between the A0-o-C1-chem-CD133 and A0-o-C2-chem groups (*p* = 0.05). The results referred to the control group K1 due to the fact that no experimental intervention was performed.

### Histological and macroscopic examination

**Figure 11:**
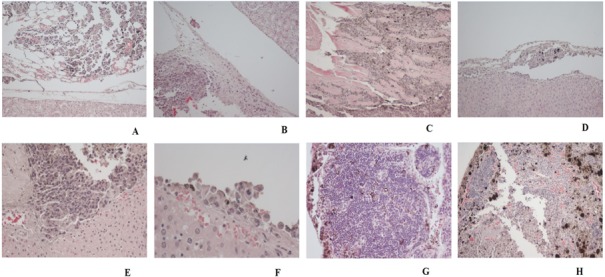
H&E staining showing the changes in different organs from all mice groups by light microscopy **A.** Focal capsule infiltration (single cells), dispersed infiltration in perirenal adipose tissue presented in a mouse kidney from an A0-o-C2-chem group. Magnification 10x. **B.** Kidney Capsule infiltration presented in a mouse from A0-o-C3-chem-CD133 group. Magnification 10x. **C.** Infiltration within the diaphragm in a mouse from A0-o-C3-chem group. Magnification 10x. **D.** Under capsule infiltration presented in mouse liver from A0-o-C2-chem group. Magnification 10x. **E.** Liver capsule infiltration presented in mouse from A0-o-C2-chem group. Magnification 10x. **F.** Liver capsule infiltration presented in mouse from control K2 group. Magnification 10x. **G.** Small foci of pleural infiltration, metastases to mediastinal lymph nodes presented in mouse from A0-o-C3-chem-CD133 group. Magnification 20x. **H.** Pleural infiltration presented in a mouse from A0-o-C3-chem-CD133 group. Magnification 20x.

The hematoxylin and eosin (H&E) staining in Figure 11 showed differences in the evolution of the metastatic and infiltration processes in each of the experimental groups. Various degrees of metastatic and infiltrating processes were observed in all animal study groups by macroscopic analysis (Figure 11). Focal capsule infiltration (single cells) and dispersed infiltration in perirenal adipose tissue presented in a mouse kidney from A0-o-C2-chem group (Figure 11A). Kidney capsule infiltration (Figure 11B) and infiltration within the diaphragm (Figure 11C) were presented in mouse from the A0-o-C3-chem-CD133 group. Under capsule infiltration (Figure 11D) and liver capsule infiltration (Figure 11E) were presented in mouse liver from A0-o-C2-chem group. Liver capsule infiltration presented in mouse from control K2 group (Figure 11F). Small foci of pleural infiltration and metastases to mediastinal lymph nodes were presented in mouse from A0-o-C3-chem-CD133 group (Figure 11G). Pleural infiltration was presented in mouse from A0-o-C3-chem-CD133 group (Figure 11H). The histological changes observed in the kidneys, lungs and liver samples of each group are presented in Table 2 with the progress of cancer expressed in terms of the lowest degree found.

**Table 1:** Control and treated groups and chemical specifications of DDS

**Study Group / DDS — description**	**Number of animals in representedgroup**
C1	5
C2	5
C3	5
AO-o-C1-chem	5
AO-o-C2-chem	5
AO-o-C3-chem	5
AO-o-C1-chem-CD133	5
AO-o-C3-chem-CD133	5
**Control Groups (K)/detailed description**	**Number of animals in represented group**
K1/without intervention	5
K2/CNT to peritoneum	5
K3/peritoneal tumors debulking	5
K4/PBS	5

**Table 2:** Histological changes observed in the kidneys, lungs and liver samples

GROUP	+/−	LIVER	+/−	LUNGS	+/−	Kidneys
C1	+	Capsule infiltrationn	-	No infiltration	+	Capsule infiltration
C2	+	Tumor foci outside the liver	-	No infiltration	+	Tumor foci outside the kidney
C3	+	Capsule infiltration	-	No infiltration	+	Capsule infiltration, diaphragmal infiltration
A0-o-C1-chem	+	Small tumor foci outside the liver	-	No infiltration	+	Capsule infiltration
A0-o-C2-chem	+	Under capsule infiltration, diaphragmal infiltration	+	Pleural infiltration	+	Focal capsule infiltration (single cells), dispersed infiltration in perirenal adipose tissue
A0-o-C3-chem	+	No capsule infiltration, tumor foci outside the liver	+	Pleura without infiltration, around the trachea tumor components	+	Extensive capsule infiltration
A0-o-C1-chem- CD133	-	No infiltration	-	No infiltration	+/−	No infiltration, separately organized group of tumor cells
A0-o-C3-chem- CD133	-	No infiltration	+	Small foci of pleural infiltration, metastases to mediastinal lymph nodes	+	Capsule infiltration
K1	+	Small tumor foci outside the liver	+	Pleura without infiltration, small tumor foci in mediastinum	+	Extensive capsule infiltration
K2	+	Capsule infiltration, in the sinuses pigment-laden cells	+	Pleura without infiltration, tumor foci in mediastinum	+	Capsule infiltration
K3	-	No infiltration	-	No infiltration	+	Focal infiltration in perirenal adipose tissue, tumor foci outside the kidney
K4	+	Capsule infiltration	-	Pleura without infiltration, metastases to mediastinal lymph nodes	+	Focal capsule infiltration (single cells)

## DISCUSSION

This study evaluated the potential of selected nanovehicles in modified targeted hyperthermic intraperitoneal chemotherapy without perfusion as a novel chemotherapeutic strategy for peritoneal carcinomatosis. Current interest in the field of experimental surgical oncology is geared towards improving standard clinical outcomes of melanoma. These generally focus on important areas such as survival rates and the quality of life [46, 47]. One important issue is how oncologic outcomes can be enhanced through the modification of current clinical procedures like HIPEC for patients suffering from PC [48]. Our *in vitro* and *in vivo* data show that novel drug delivery systems represented by nanovehicles (nanocontainers with the selected cytostatics and supported by a specific antibody) have a great deal of potential in the treatment of melanoma.

Such an innovative approach could be clinically more beneficial and effective in the palliative treatment of PC than the HIPEC method that is currently employed. Several recent *in vitro* and *in vivo* studies on anticancer strategies based on novel drug delivery tools and targeted therapy mechanisms have been reported [49]. Previous studies have also shown that carbon nanotubes are good delivery tools, which can control the bioavailability of a drug depending on its location [34]. Recent strategies for treating cancer have utilized antibodies that have been directed against specific surface antigens or receptor proteins within tumor cells. This strategy has proven efficient at targeting drugs or their carriers to the specific area requiring treatment [50]. DDS could prove more effective in chemotherapy than HIPEC due to the prolonged drug release properties. Current HIPEC treatment procedures are characterized by intraperitoneal perfusion of 1-3 hours in one intraoperative session [51, 52]. This results in limited clinical effectiveness due to the relatively short time that cancer cells are exposed to cytostatic drugs, explaining why some patients also later qualify for standard systemic chemotherapy. Another related problem is that not all patients in the relevant clinical condition qualify for multiple HIPEC interventions [53]. Melanoma is not the most common cause of PC in humans. It is most commonly associated with ovarian cancer, malignant mesothelioma, benign papillary mesothelioma and desmoplastic small round cell tumors [54, 55].

The anticancer properties of DDS against melanoma cells were previously described [56, 57]. In order to construct a novel DDS with anticancer properties, standard anticancer drugs were chosen such as Pt(II) complexes. The efficacy of CDDP has been determined by evaluating the chemosensitivity of melanoma cells [58,59]. Prodrugs were also introduced free or bound to nanotubes using physical or chemical methods, providing a more flexible approach. The cytotoxicity screening of all the particular elements (Pt-complexes, nanotubes) as well as the drug delivery systems was performed on normal rodent CHO cells. Both C2 and C3 Pt-complexes are more toxic than the C1 variant whereas the nanotubes themselves (A0-o) seem to be nontoxic even when applied at the highest doses. These observations suggest that A0-o systems could be used for novel DDS applications for cancer treatment. The chemical bonding of Pt-complexes to the nanotube surfaces failed to induce any significant alterations in their toxicity whilst the A0-o-C3-chem system proved most toxic. Of all complexes, only C2 acted in a different manner from the rest in that its use resulted in excessive cell membrane damage. Overall, the effectiveness of the new drug delivery systems indicates that this novel approach is a useful therapeutic direction to follow in attempting to treat this type of melanoma. The data demonstrates that nanotubes can be used to effectively deliver therapeutics to the nucleus of a cell while the large drug delivery systems were unable to enter the cell and therefore exhibited reduced cytotoxicity. This effect is more pronounced in normal cells since their proliferation rate was much slower than in cancer cells.

Another important aspect of the data was how the anti-CD133 antibody was successfully attached to nanotubes. Flow-cytometry analysis showed that nanotubes connected to the antibody have the ability to target cells expressing CD133 antigen and can potentially be used in experimental therapy against cancer stem cells. CD133-positive melanoma cells are phenotypically characterized as melanoma cancer stem cells [60, 61], which are resistant to chemotherapy and responsible for disease reoccurrence [62]. Differences were also noted between slow-acting and fast-acting DDS during the *in vitro* experiments on B16 cell lines: the LT50 was found to be shorter in the case of samples obtained using physical methods. Real-time cell analysis also showed that drugs administered alone and those delivered physically with nanotubes acted similarly, whilst there was a slower decrease shown in the proliferation rate when chemically modified nanotubes were employed. This phenomenon can be explained by the gradual release of drugs after chemical modification leading to a prolonged effect on cell proliferation. Overall, this data suggests that treatment with the new DDS increases the effectiveness of Pt-based drugs and could lead to an improvement in the efficiency of the anticancer treatment.

The *in vivo* study showed the shortest survival rate in the control group (K3) where no hyperthermic intraperitoneal chemotherapy procedures were used and surgical cytoreduction of tumor masses was performed. This result could be due to two factors: First, the surgical procedure was not accompanied by any type of adjuvant and neoadjuvant chemotherapy, Second, the standard “Sugarbaker's protocol” and its numerous modifications assume the implementation of cytoreduction only in groups of patients who meet all qualification criteria for HIPEC [63, 64]. In this study, cytoreduction was performed on all animals from group K3 in the absence of a Peritoneal Cancer Index (PCI) or any exclusion criteria such as that applied to those with a substantially large tumor that possibly resulted in a higher mortality rate [65]. On the other hand, the longest survival median rate was observed in the group A0-o-C1-chem. That may due to the fact that the drugs that were used for *in vitro* tests exhibited high levels of activity and caused a lot of side effects including killing healthy cells. Therefore, in order to create effective DDS with minimal side effects, it is necessary to use slow-acting systems functionalized with targeting moieties i.e. DDS with antibodies and conjugated prodrugs. Overall, the main benchmark from analysis of the results was found in the survival rate of the control group (K1) where no experimental procedures were performed after the implantation of the B16 melanoma cells. It is striking that this group had longer survival rates versus many of the groups where experimental procedures were carried out. This is likely due to their invasive nature as well as complications associated with surgery and chemotherapy. Many of these approaches are used to relieve pain, and improve the quality of life and cannot be evaluated or compared when using the small rodent model [66,67].

The most important aspect of this study is findings that carbon nanotubes can be used as DDS in light of ongoing controversy in several publications suggesting that carbon nanotubes themselves exhibit a high level of cytotoxicity [57, 58]. Our study suggests to the contrary that cytotoxicity is observed when nanotubes are used either *in vitro* or *in vivo*. The manifestation of CNT cytotoxicity could be explained by the presence of residual metallic catalysts in nanoparticles that were observed mainly in cell culture studies [68-70]. In summary, we tested novel drug delivery systems based on carbon nanotubes loaded with Pt-prodrugs and functionalized with anti-CD133 antibodies. The DDS have been found to be useful tools in targeted anti-cancer therapy in both *in vitro* and *in vivo* studies. We found most effective planned and targeted palliative therapy on experimental melanoma peritoneal carcinomatosis using animal model. Our multidisciplinary studies will demonstrate the utility of a novel form of therapies dedicated to patients suffering from peritoneal carcinomatosis.

**Figure 12:**
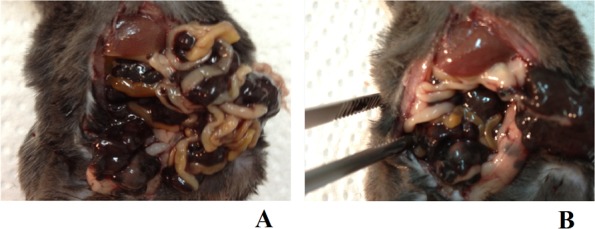
Sample macroscopic pictures from autopsy **A.** A mouse from the A0-o-C1-chem-CD133 group with metastatic PC in the peritoneal cavity. **B.** A mouse from the K1 group with metastatic PC in the peritoneal cavity after the partial removal (during dissection/post-mortem) of large tumor masses which covered the visceral organs.

## MATERIALS AND METHODS

### Nanotubes and drug deposition

**Scheme 1:**
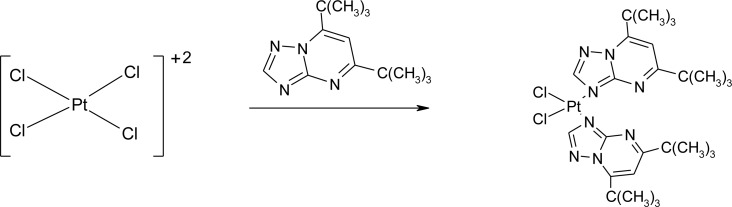
Outline of C2 synthesis

**Scheme 2:**
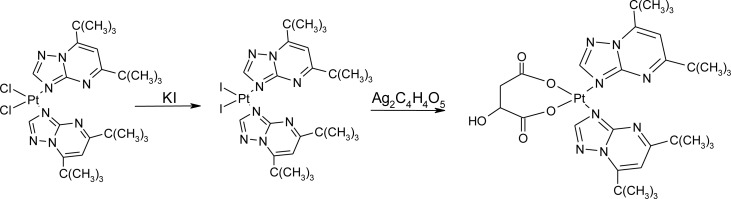
Outline of C3 synthesis

Open-ended high-purity single-walled carbon nanotubes (labeled A0-o) were obtained from Nanostructured & Amorphous Materials (NanoAmor, Houston, TX, USA). These were hydrothermally oxidized using 30% H_2_O_2_ at 493 K temperature as previously described [34, 35]. C2 was synthesized by reaction of K_2_PtCl_4_ with dbtp at a molar ratio of M:L = 1:2 as outlined in scheme 1 [36]. Reaction of C2 with Ag_2_C_4_H_4_O_5_ [Pt(C_4_H_4_O_5_)(dbtp)_2_] was performed to obtain C3 as shown in scheme 2 [36]. Both coordination compounds had previously been structurally characterized by IR; ^1^H, ^13^C, ^15^N, ^195^Pt NMR, and single-crystal X-ray diffraction [37, 38].

**Scheme 3:**
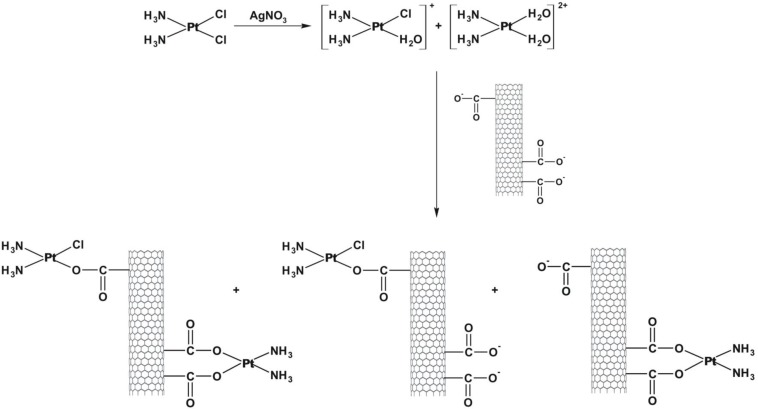
Outline of A0-o-C1-chem synthesis

The C1, C2 and C3 complexes were then introduced to the nanotubes by either physical deposition through a simple evaporation process or chemically bonded. As outlined in scheme 3, the synthesis of A0-o-C1-chem was achieved using a modified procedure previously described by *Ye et al* [39]. The C1 complex (20mg) was added to 20 ml of water and mixed with 11.8 mg of silver nitrate to form an aqueous complex [36]. The resulting AgCl precipitate was centrifuged at a speed of 8000xg and the supernatant was filtered twice through a 0.22μm filter before being attached to the carbon surface A0-o sample (75 mg) dissolved in 1 ml of isopropanol. To achieve a slightly alkaline environment, several drops of 0.1M NaOH were added to the purified supernatant. The solution was then mixed vigorously for 24 hours at temperature of 310K. Next, the mixture was filtered through a 0.8μm filter (the sample labeled A0-o-C1-chem-n, where n means “not washed”) and washed with DMF (3×10 ml). The products were dried in a vacuum chamber at a temperature of 323 K.

**Scheme 4:**
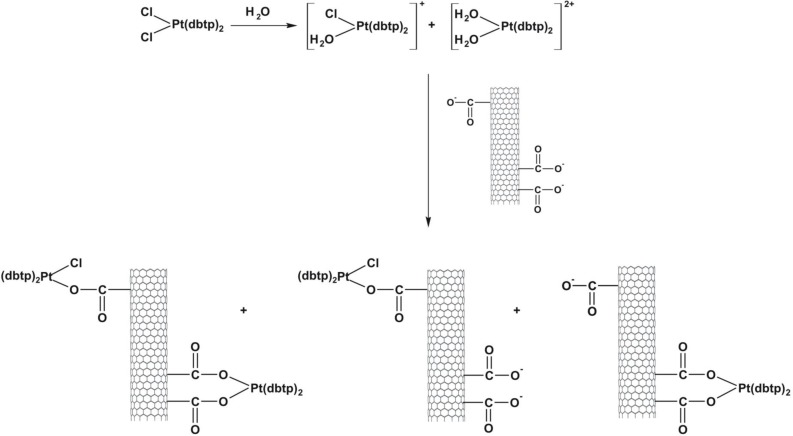
Outline of A0-o-C2-chem synthesis

**Scheme 5:**
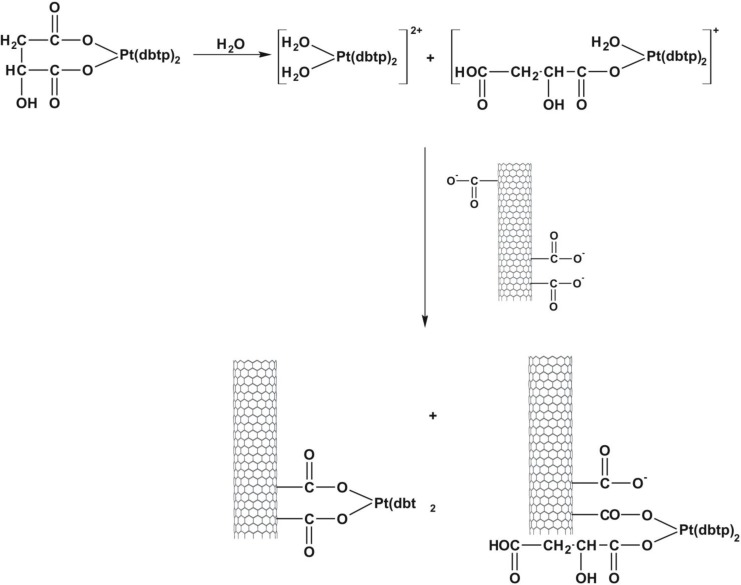
Outline of A0-o-C3-chem synthesis

*Cis*-diamminedichloroplatinum C1 (CDDP, Sigma–Aldrich, 99.9% pure) was deposited as previously described [40]. A sample of A0-o-493 (30 mg) was dispersed (by sonication) into a solution of CDDP (60 mg) in water (10 ml) and vigorously stirred for about 20 hours at room temperature. Next, the solvent was removed by evaporation in a vacuum chamber at 323K. The resulting sample was labeled as A0-o-C1–phys and had a CDDP: nanotube mass ratio of 2:1. The physical deposition of C2 was performed as follows: an A0-o sample (30 mg) was dispersed by sonication in a solution of C2 (60 mg) in10 ml of ethanol (or in10 ml of acetone in the case of C3) and stirred for about 20 hours at room temperature. The organic solvents were used to prevent the hydrolysis of the Pt-complexes. Next, the solvent was evaporated using a vacuum chamber at a temperature of 323K to yield of A0-o-C2 (/C3)-phys with C2(/C3):nanotube mass ratio of 2:1. To attach the C2 (/C3) complex to the carbon surface, a solution of A0-o sample (75 mg) in 1 ml of isopropanol was used to fill the carbon nanotubes. The synthesis of A0-o-C2- and A0-o-C3 are outlined in schemes 4 and 5. Following this, the sample was dispersed by sonication in a mixture of water (15 ml) and several drops of 0.1M NaOH added gradually to 20mg of C2 (/C3) to obtain a slightly alkaline solution. These conditions forced both reagents, i.e. oxidized CNT and C2 (/C3) complex to be hydrolyzed. The resulting solution was vigorously stirred for about 44 hours at room temperature. The mixture was then filtered through a 0.45 μm filter (the sample labeled A0-o-C2 (/C3)-chem-n, where n means “not washed”) and washed with DMF (in the case of C2; 3 × 10 ml) or acetone (in the case of C3; 3 × 10 ml) before being dried in a vacuum chamber at temperature of 323K to give A0-o-C2 (/C3)-chem.

### Preparation of A0-o-CX-chem-CD133 complex

The synthesis of A0-o-CX-chem-CD133 was achieved using a multistep process. First, a sample of A0-o-CX-chem (10 mg) was dispersed (via sonication) in 10ml of PBS and then stirred for about one hour at a temperature of 277 K to attach the BSA (10 mg) to the CNT. Next, the sediment was centrifuged, washed with PBS (3 times) and dispersed via sonication. EDC (38 mg) and NHS (58 mg) were then added and the mixture was stirred for approximately one hour at a temperature of 277 K. The resulting mixture was dispersed (via sonication) in 5 ml of PBS, followed by addition of anti-CD133 antibody at concentration of 0.2 mg/ml (Biorbyt LLC, USA). The mixture was then stirred for 2 hours at 277 K and centrifuged. The resulting sediment was washed with PBS (3 times) to yield the final product of A0-o-CX-chem-CD133, which was stored at a temperature of 277 K in PBS.

### Electron microscopy and thermal analysis

High-resolution transmission electron microscopy (HRTEM) images were taken using a transmission electron microscope F20X-TWIN (FEI-Tecnai). In addition, Z-contrast imaging was done with a scanning TEM equipped with a Fischione HAADF (High Angle Annular Dark Field) detector. Both devices operated at a voltage of 200 kV. For thermogravimetric measurements of the atmosphere, simultaneous TGA-DTA Thermal Analysis TA Instruments (SDT 2960) was used at a temperature range of 293-1073 K at a heating rate of 10K/min. Nitrogen adsorption isotherms were measured using an ASAP2010 volumetric adsorption analyzer from Micromeritics (Norcross, GA), which was operated at the temperature of liquid nitrogen (77K) and at pressures ranging from about 10^−6^ to 0.999. The A0-o-C1 samples were degassed for 2 hours at temperature of 473K whilst the samples loaded with C2 and/or C3 were heated at 373K.

### *In vitro* cytotoxicity and proliferation assays

Cytotoxicity screening of A0-o loaded with Pt complexes was carried out using CHO (Chinese hamster ovary) cells. These are rodent epithelial adherent cells obtained from Sigma-Aldrich and cultured according to the manufacturer's protocol. These cells are used regularly as mammalian cell models in biological, medical and pharmaceutical research [41, 42]. Approximately 5×10^4^ cells were seeded into each well of a 12-well plate for 24 hours and grown in F-12 medium (containing 10% fetal bovine serum (FBS), 100 μg ml^−1^streptomycin and 100 U ml^−1^ penicillin) in an incubator with 5% CO_2_ at temperature of 309K. CHO cells were treated with each of Pt complexes at concentrations of 1, 10, and 50μg ml^−1^ and tested in time-dependent manner from 24 to 72h.

The effect of DDS on cell growth and viability was then examined in triplicate through MTT and LDH activity assays. The MTT solution was prepared at a concentration of 1 mg ml^−1^ in F-12 medium and then added into each well containing the CHO cells. Following an incubation period of 15 min, the solution was discarded and a solution of Formosan crystals dissolved in DMSO was added and their absorbance measured at a wavelength of 570nm. LDH was then employed to convert pyruvate to lactate in the presence of NADH as a proton donor and its level of activity measured within a culture medium by the observable decrease of NADH into its oxidized form of NAD (β-nicotinamide adenine dinucleotide). The absorbance was measured at a wavelength of 340 nm using a spectrophotometer and directly correlated with the increase in the number of damaged cells. For the LDH activity assay, 100 μl of NADH (1.25 mg ml^−1^) and 100 μl of sodium pyruvate (2.5 mg ml^−1^) were added into 600μl of culture medium before the number of living and damaged cells was compared to the control sample.

Mouse melanoma cells B16-F10 (CRL-6475™) were purchased from the American Type Culture Collection (ATCC, Manassas, VA) and cultured in DMEM/HAM's F-12 medium containing 10% of FBS supplemented with 5-μg ml^−1 of^ amphotericin B, 100-μg ml^−1^ of streptomycin, and 100 U ml^−1^ of penicillin (pH 7.4). The cells were grown in 25 cm^2^ flasks (PAA, Austria) with 5% CO_2_ atmosphere at a temperature of 309K. The effect of DDS on tumor cells was then measured using the Real Time Cell Analyzer (RTCA) (x-CELLigence system, Roche Applied Science), which enabled cellular events to be monitored in real time. The cells (*n* = 4000) were subsequently seeded onto all the wells of an E-Plate 16 (Roche Applied Science) and incubated in cell culture medium for 20 hours. Varying concentrations of DDS (10, 50, 150 and 250 μg ml^−1^) were then added and incubated for an additional 72 hours. Viability was expressed in cell index (CI) values. The increase in electrode impedance on an E-Plate due to the increase in cell numbers attached to the electrodes was used to monitor the viability, number, morphology, and the degree of adhesion within a number of cell-based assays. The experiment was repeated three times with isolated cells with a cell-free medium serving as control. The average curves plotted in Figure 7 displays the relative values compared to the control and were calculated using the method described by Werengowska-Ciećwierz et al [40].

### Flow cytometry analysis

Cells were isolated from the peripheral blood of a hematopoietic donor that had been stimulated with high dose G-CSF (Granulocyte-Colony Stimulating Factor) (amounting to 10 mcg/kg of filgrastim per day over 4 days). Mononuclear cells were separated from the peripheral blood by apheresis using the cell separator Cobe^®^Spectra (Terumbo BCT, USA). The isolated cells contained mainly lymphocytes and monocytes and a large amount of hematopoietic cells together with platelets and a small number of erythrocytes.

The cell pellets were resuspended in ice-cold FACS Buffer (PBS with 5% FBS) and incubated for one hour in the dark at a temperature of 4°C with the FITC-labeled anti-CD133 antibody connected to nanotubes. The cells were washed 3 times by centrifugation and resuspended in 500μl of ice-cold FACS buffer and maintained in this state until CD133 expression analysis was performed using FACSCanto flow cytometry (BD Bioscience, USA).

### Peritoneal carcinomatosis animal model

Ten weeks old C57BL/6J mice were obtained from Charles River strain. Animals were divided into 12 groups (where *n* = 5 in each group): four control groups and eight study groups (labeled as: C1, C2, C3, A0-o-C1-chem, A0-o-C2-chem, A0-o-C3-chem, A0-o-C1-chem-CD133, A0-o-C3-chem-CD133) were designed to test all new nanovehicles. Table 1 shows the details of treated and control groups as well as the type of the DDS employed. All animal procedures followed guidelines approved by the Ethical Committee of official European Union recommendations and guidelines (2010/63/EU) of animal use.

To artificially create peritoneal carcinomatosis, B16 mouse melanoma (CRL-6475™) cells were used. The B16 cells were cultured according to the manufacturer's conditions in 25-cm^2^ Nunc T-flasks at a temperature of 309K in an atmosphere containing 5% CO_2_ and 95% humidity until the 3^rd^ passage. About 1 × 10^6^ cells were injected into the lower-right quadrant of the abdomen (Figure 1). Injections were performed in a rigorously consistent manner at the exact same time each day after the surrounding area had been disinfected.

### Experimental chemotherapy using PC animal model

Melanoma cells were implanted for a week after which the PC process could be clearly observed. Hyperthermic intraperitoneal chemotherapy was administered without perfusion using the range of DDS across all eight-study groups. Before the DDS were administered, they were sonicated in 0.7 ml of sterile PBS for 3 minutes to obtain a homogenous solution. It is believed that this volume of PBS is the optimum amount, which could be safely applied to the peritoneal cavity for any period of time and constitutes approximately 25-29 % of the total blood volume (TBV) in mice, to reduce postoperative complications [43]. To replicate the clinical standards of HIPEC treatment without perfusion, a previously prepared solution containing chemotherapeutic DDS was also heated to the average HIPEC solution temperature of 43°C [44, 45].

After disinfecting the operating area and inducing anesthesia, a midline incision along the *linea alba* was performed on all animals in the study groups. Following preparation of the skin, muscles and other tissues, the abdominal cavity was exposed as shown in a Figure 1. Employing the classic open method of hyperthermic intraperitoneal chemotherapy, a Castroviejo eye speculum was used to hold the abdominal wall open and standard surgical sutures were used to close off the surgical area after treatment (Figure 1).
